# Association of sleep quality, media use and book reading with behavioral problems in early childhood. The Ulm SPATZ Health Study

**DOI:** 10.1093/sleepadvances/zpac020

**Published:** 2022-06-24

**Authors:** C Ricci, T Poulain, J Keil, D Rothenbacher, J Genuneit

**Affiliations:** Pediatric Epidemiology, Department of Pediatrics, Medical Faculty, Leipzig University, Leipzig, Germany; LIFE Leipzig Research Center for Civilization Diseases, Leipzig University, Leipzig, Germany; Department of Women and Child Health, Hospital for Children and Adolescents and Center for Pediatric Research (CPL), Leipzig University, Leipzig, Germany; Department of Child and Adolescent Psychiatry, Psychotherapy and Psychosomatics, Medical Faculty, University of Leipzig , Leipzig, Germany; Institute of Epidemiology and Medical Biometry, Ulm University, Ulm, Germany; Pediatric Epidemiology, Department of Pediatrics, Medical Faculty, Leipzig University, Leipzig, Germany; Institute of Epidemiology and Medical Biometry, Ulm University, Ulm, Germany

**Keywords:** sleep quality, media use, book reading, behavior, early childhood, SPATZ study

## Abstract

**Study Objectives:**

The objective of the study was to investigate the association of sleep quality, media use and book reading on internalizing, externalizing and prosocial behavior in early childhood.

**Methods:**

In this cross-sectional study, we investigated a data set consisting of three consecutive yearly waves of the prospective Ulm SPATZ Health Study, conducted in southern Germany with 565, 496, and 421 children of 4–6 years of age, respectively.

Standardized effects of the overall score and subscales of the Children’s Sleep Habits Questionnaire, parent-reported child media use and book reading as well as their interaction term on the total score of the Strengths and Difficulties Questionnaire along with its externalizing, internalizing and prosocial subscales were estimated by multivariate adjusted random intercept mixed models.

**Results:**

Overall sleep quality was associated more with internalizing than externalizing behavior; parasomnias associated with both behaviors. Night waking and sleep anxiety associated only with internalizing behavior. High levels of media use were associated with less internalizing behavior. More book reading resulted in less externalizing and internalizing behavior but more prosocial behavior. Finally, book reading and media use do not interact to determine child’s behavior.

**Conclusions:**

The current work supports a strategy of monitoring sleep quality, reducing media use and promoting book reading in order to avoid behavioral problems in early childhood.

**Statement of Significance**

Low sleep quality and high levels of media use negatively influenced the behavior of German children from 4 to 6 years. On the contrary, book reading was related to better prosocial behavior and less externalizing and internalizing behavior. Thus, suitable strategies to improve the behavior of young children that age include the monitoring of sleep quality, a reduction of media use or the substitution of media use with book reading.

## Introduction

In Germany, behavioral problems in children represent a public health threat leading to increased healthcare use and considerable economic costs. For example, studies show that up to 50% of German school children may have some type of behavioral problem corresponding to increased medical costs of about 300 € per child every year [1]. Externalizing behaviors are maladaptive behaviors directed toward an individual’s environment and peers. These include problems such as disruptive behavior, attention-deficit or hyperactivity, conduct disorder and oppositional defiant disorder [2]. Numerous factors may lead to an externalizing behavior in children. Particularly, the role of parent–child interaction, lack of effective guidance and parental ego-resiliency emerged in previous studies as being possible externalizing triggers [3]. With internalizing behaviors, an individual internalizes or keeps inside maladaptive emotions. These include depression, anxiety, dissociation, obsessive-compulsive, and related behavior [4]. Such internalizing behavior and reduced prosocial attitude might be related to a lack of positive social exploration related to nonadequate guiding, children’s perception of insecure, socioecological, and personal-social conditions [5]. Both, externalizing and internalizing problems, are commonly observed in children from developed countries [6–8] resulting in an increasing demand of studies to investigate the determinants of such behavioral problems in school children.

So far, some empirical studies have investigated the influence of TV watching, media use, book reading and sleep quality on mental health in children. These studies demonstrated that media use and TV watching are strong predictors of externalizing problems in children [9]. In contrast, children who read more books displayed less externalizing and internalized problems [10]. Furthermore, prior work shows that a low sleep quality increased the risk of both internalizing and externalizing problems in children and also adolescents [9, 11–14]. Moreover, there is evidence that sleep problems occurring in early childhood can detrimentally impact the mental health of those children in later life [15–17]. For instance, according to findings from a longitudinal study, behavioral and emotional problems in mid-adolescence can be predicted by sleep problems at the age of 4 [16]. Hence, this association between sleep quality and problematic behavior seems to be directional, which also supports Dahl’s and Walker’s model of sleep in relation to emotional functioning and possibly to behavior [18, 19]. Interestingly, some studies indicate that specific sleep problems differentially affect problematic behavior in children [12, 20, 21]. For instance, while a reduced sleep duration is related to externalizing behavior, irregularity in sleep duration may rather lead to internalizing problems [21].

Furthermore, studies also showed that book reading and media use can impact sleep quality [22–26]. For instance, an observational study demonstrated increased sleeping duration, better sleep quality as well as efficiency in young children who frequently read a book before bedtime. On the contrary, sleep quality was impaired in children who watched TV during the hour before sleep [25].

Given the little amount of studies on the relationship of book reading and sleep quality in general, it is especially interesting to investigate of whether book reading might be beneficial for sleep quality in early childhood and, further, if book reading could compensate for potential problematic behavior resulting from low sleep quality. Moreover, there is also a paucity of knowledge of how media use and book reading possibly interact to affect behavioral problems or whether these factors act rather independently. By including these interaction terms we aimed to examine to what extent book reading may represent a better alternative to media use to entertain young children by, for instance, compensating problematic behavior related with high media use.

Furthermore, despite this aforementioned evidence in middle-aged children, we do not know whether and how these effects unfold in early childhood. Thus, it is of great importance to investigate behavioral problems in younger children to either prevent their onset or lower the risk of aggravation of these problems at school, during adolescence or up to adulthood [27].

The aims of our work are therefore manifold. First, we explored how sleep interacted with media use or book reading, determining child’s behavior as a whole, along with internalizing, externalizing and prosocial behaviors. We further aimed to explore how the interaction between media use and book reading influenced child’s behavior in early childhood.

## Methods

### The Ulm SPATZ Health Study

The Ulm SPATZ Health Study is an ongoing prospective birth cohort study at the University Medical Center Ulm (Southern Germany) in which 1,006 children were consecutively recruited after their birth between April 2012 and May 2013. Data at baseline were collected shortly after the children’s delivery and consecutively every birthday. Participating families were asked to fill in a battery of self-administered written questionnaires. The present work includes data from study waves 6, 7, and 8 at the children’s ages of 4, 5, and 6 years, respectively. Informed consent was obtained from all mothers and the study was approved by the Ethics board of Ulm University (No. 311/11).

## Assessment of Behavioral Strengths and Difficulties

The German version of the Strengths and Difficulties Questionnaire (SDQ) was administered to caregivers for quantifying child behavior. The SDQ consists of 25 items (3-point scale) resulting in five symptom scales (conduct problems; hyperactivity; emotional symptoms; peer problems and prosocial behavior). For this study, the conduct problem and hyperactivity subscales were summed to compute an externalizing behavior score while the emotional and peer problem subscale summed to yield an internalizing behavior score [28]. The prosocial behavior subscale was considered separately. Thus, higher externalizing and internalizing behavior scores correspond to poorer mental health, while higher scores of the prosocial behavior subscale mirror more adaptive psychosocial functioning of children. The SDQ was validated in the German population [29].

## Assessment of Children’s Sleep Quality

To assess children’s sleep quality, parents filled in the validated German version of the Children’s Sleep Habits Questionnaire (CSHQ) [30, 31]. The CSHQ is a multidimensional questionnaire comprising 34 items (3-point-scale as “Usually”, “Sometimes”, and “Rarely”) yielding eight sleep quality subscales (bedtime resistance, sleep onset delay, sleep duration, sleep anxiety, night waking, parasomnias, sleep-disordered breathing, daytime sleepiness). For the current analysis, each subscale and the total CSHQ score were used. The total score was computed by the sum of all 34 CSHQ items with higher scores representing lower sleep quality.

## Assessment of Digital Media Use and Book Reading

To assess the quantity of digital media use and book reading, parents were asked to document their childrens’s average duration of electronic media use and book reading separately during week days and weekends using a time spacing scale where items were coded as: never; up to 1 h/day; 1 to <2 h/day, 2 to <3 h/day, 3 to <4 h/day and ≥ 4 h/day. That weekends and week days were analyzed separately was motivated by findings showing that the time spent by children in using digital media differs between the two [32].

Time spent watching TV or DVDs as passive activities in front of a screen and active use of a computer, tablet or smartphone were summed to compute the quantity of digital media use. A second variable reflecting the quantity of book reading was computed by the sum of time spent in self or parent assisted book reading.

## Statistical Methods

Behavioral scores and subscales, sleep scores and subscales, time spent with media use and book reading during week days and weekends were described using median, 10th and 90th percentiles at different SPATZ waves.

However, to ensure that these variables did not change over the observational time, we performed a kernel nonparametric regression having the behavioral score, the sleep score and time spent with media use and book reading during week days and weekends as outcome while time was the explanatory covariate. Model fitting from nonparametric analyses of the trend over time was reported by means of nonparametric analogue *R*^2^ [22] representing variance explained by time (Table 1). As there was limited evidence for time-dependent changes, data of the three consecutive waves were aggregated to increase the statistical power for the following main analysis.

**Table 1.** Median, 10th and 90th percentiles of behavior scores and subscales, sleep scores and subscales and media use during week end week-ends use in SPATZ study at age 4, 5, and 6 years

	Age 4 year old *N* = 565	Age 5 year old *N* = 496	Age 6 year old *N* = 421	* Time trend *R*^2^ (%)
*Behavior scores and subscales*				
Internalization score	4 (1, 8)	3 (0, 8)	3 (0, 7)	0.9
Emotion	1 (0, 3)	1 (0, 4)	1 (0, 3)	0.07
Behavior	2 (0, 4)	2 (0, 4)	1 (0, 4)	0.8
Externalization	2 (0, 4)	2 (0, 4)	1 (0, 4)	0.1
Hyperactive	3 (0, 6)	2 (0, 6)	2 (0, 5)	0.5
Peers	1 (0, 3)	1 (0, 3)	0 (0, 3)	0.7
Prosocial	8 (5, 10)	8 (5, 10)	8 (6, 10)	0.8
SDQ	7 (2, 14)	6 (2, 13)	6 (1, 12)	0.7
*Sleep disturbance score*				
Bed difficulty	8 (6, 12)	7 (6, 12)	7 (6, 12)	0.07
Delay in falling asleep	1 (1, 2)	1 (1, 2)	1 (1, 2)	0.001
Sleep time	3 (3, 5)	3 (3, 5)	3 (3, 5)	0.03
Sleep-related fears	6 (4, 8)	5 (4, 8)	5 (4, 8)	0.5
Waking up at night	4 (3, 7)	4 (3, 7)	3 (3, 7)	1.6
Parasomnia	7 (6, 9)	7 (6, 9)	7 (6, 9)	0.07
Sleep-related breathing disorders	3 (3, 4)	3 (3, 4)	3 (3, 4)	0.2
Daytime sleepiness	11 (9, 15)	11 (9, 15)	11.5 (8, 16)	0.001
Total sleep disturbance score	43 (37, 53)	43 (36, 52)	42 (36, 52)	0.4
*Media use and book reading (minutes)*				
Media use during week	30 (0, 90)	30 (0, 90)	30 (0, 120)	0.4
Media use during week ends	42 (0, 120)	60 (12, 120)	60 (12, 159.6)	3.1
Book reading during week	60 (24, 120)	42 (12, 102)	54 (12, 120)	0.3
Book reading during week ends	60 (12, 120	60 (12, 120)	60 (12, 120)	0.05

*R^2^ model’s goodness of fit for Kernel nonparametric regression having time as covariate.

For the main analysis, the SDQ behavioral scores and subscales, CSHQ sleep quality score and subscales, time spent with media use or book reading were rescaled to sex-specific normalized *Z*-scores using Blom’s inverse rank transformation [33]. Subsequently, we investigated the relation between sleep quality, media use and book reading scores and internalizing and externalizing as well as prosocial behavior using a random intercept mixed model adjusted for baseline maternal age, maternal education and Blom’s normalized *Z* scores of maternal anxiety and depression scores assessed by the German version of the Hospital Anxiety and Depression Scale [34]. Specifically, subject (at 4, 5, and 6 years of age) was treated as random effect while all the other covariates were considered as fixed effects.

The strength of the association of the SDQ score and subscales with the CSHQ sleep quality score and subscales, media use or book reading during week days and the weekend and their interaction term was reported using student-*t* metric of the statistical effect size (*t*-ES = |slope/stderr|). Using this metric, the threshold value of 1.96 could be assumed as statistically significant (α = 0.05, type-I error = 5%). Our work was exploratory in nature, but to give insight into required account for multiple testing in a hypothesis testing framework, Bonferroni correction was applied (α = 0.00625).

Forrest plots were performed plotting standardized coefficients and their 95% confidence limits. Notably, following the standardization of the outcome, these statistical effects are interpretable as standardized effect sizes with values in the range 0–0.2, 0.2–0.5 and above 0.5 as small, medium and large effect sizes, respectively. All analyses were performed with the total SDQ score as well as internalizating, externalizating, and prosocial behavior subscales as main outcomes. Supplementary evaluations were conducted considering the original four emotional, behavior, hyperactive and peers subscales. Supplementary analyses adjusting for paternal characteristics (age and education), for characteristics of both parents (smoking and drinking) and considering birth season as well as diet of the children have been conducted to evaluate potential residual confounding. Indicator variables were used in supplementary analyses to account for missing values due to additional covariates. All analysis on media use and book reading were mutually adjusted so that both features are included in the model. Finally, a further analysis was conducted to investigate the interaction between media use and book reading on the week and the weekend. This last analysis was based on the same mixed model above described and was further adjusted for overall CSHQ score. Sensitivity analyses were performed comparing outcome results between the total sample and the analytical sample for which all covariates were coded.

All statistical analyses were conducted using the R statistical software. Nonparametric kernel regression for trend over time was performed using the NP package, the mixed model analyses were performed using the LME4 package.

## Results

### Sample characteristics

In general, an aggregated data set of the three waves of SPATZ (with children aged 4, 5, and 6 years) was used with 565, 496, and 421 records, respectively. The sample was balanced for child sex with boy to girl ratio ranging between 0.98 (at wave 8) to 1.03 (at wave 7). The baseline age of the mother was on average 33 years, ranging between 21 and 54 years; maternal education was high with approximately 70% of the mothers having at least a high school degree. Note, this high maternal education was representative of the geographical area of the SPATZ data set [35].

All main variables under investigation remained relatively constant with less than 2% variability explained by observational time (Table 1). Overall, the sample had SDQ scores in the normal range according to the normative data for German children of higher age and Australian children of the same age [25, 26].

More time per day was spent on media use and book reading during the weekend than week days and irrespectively of child age. Time spent per day with book reading during the week or weekend and media use during the week were constant during the observational period. On the contrary, we report an increased use of media use per day during the weekends between 4 and 6 years of age, with time explaining about 3% of the observed variability.

### Associations between sleep quality, media use, and behavior

Regarding the relation between sleep quality, media use and the total SDQ score, we observed significant associations with sleep anxiety, parasomnias, day time sleepiness and the overall CSHQ sleep quality score. However, media use itself was not related to the total SDQ score.

Notably, media use and sleep quality scores appeared as independent since none of the interaction term between sleep quality scores and media use was statistically significant.

**Figure 1.**
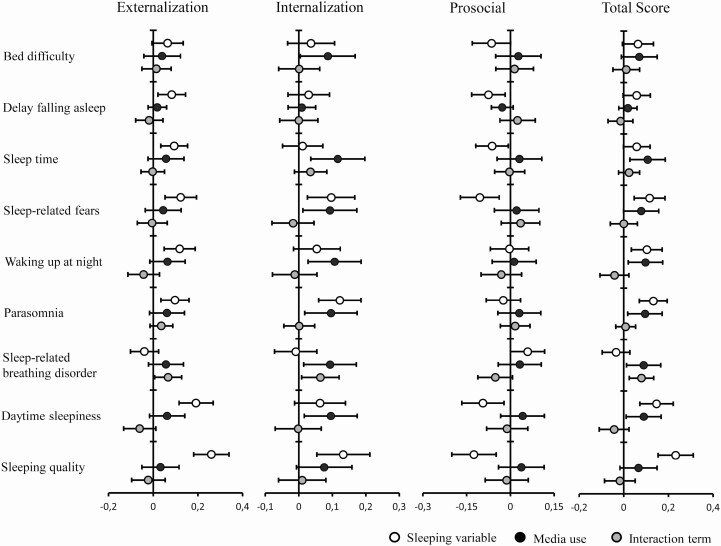
Estimates and 95% CI of standardized beta coefficients of behavioral scores in relation to media use during the week.

**Figure 2.**
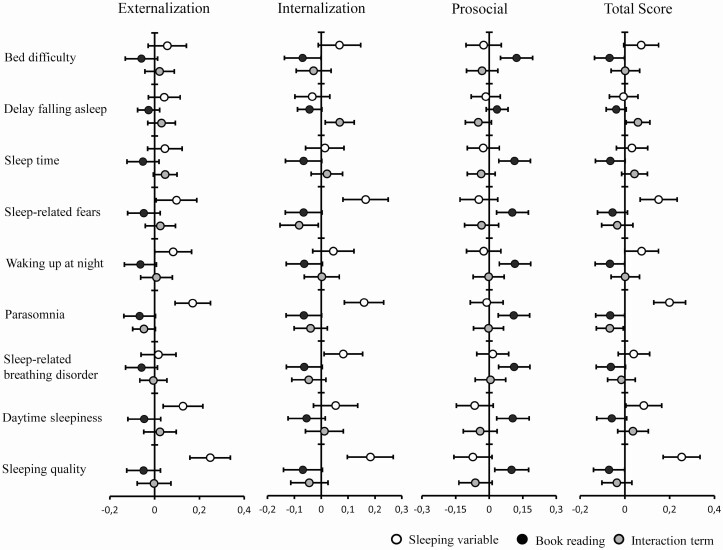
Estimates and 95% CI of standardized beta coefficients of behavioral scores in relation to book reading during the week.

When considering externalization and internalization subscales of the SDQ, we confirmed that sleep quality is associated with both externalizing and internalizing behaviors. Specifically, when considering the analyses conducted for week days, the overall sleep quality score and its subscales of sleep duration, sleep anxiety, night waking, parasomnias and day time sleepiness were related more to the internalizing behavior score (*t*-ES = 3.0, 3.0, 2.7, 3.0, 2.6, and 7 for sleep duration, sleep anxiety, night waking, parasomnias, day time sleepiness subscales and total CSHQ score, respectively). The parasomnias subscale along with total CSHQ score were strongly associated to the externalizing symptom score (*t*-ES = 5.5 and 4.0 for parasomnias subscale and total CSHQ score, respectively). Finally, daytime sleepiness and the overall CSHQ scores were negatively associated to lower prosocial scores while media use did not influence it (*t*-ES = 3 for both daytime sleepiness subscale and the CSHQ score). A summary of the results are displayed in Figures 1 and 2.

### Associations between sleep quality, book reading, and behavior

There were no significant interaction effects between book reading and sleep quality with regard to prososcial, externalizing as well as internalizing behavior. Both, sleep quality and book reading are associated with these behaviors independently, hence, neither in an antagonistic nor an synergistic way.

In particular, when looking at the association between book reading and SDQ score and subscales, a beneficial role of book reading arose. More specifically, reading more books was associated with lower levels of externalizing and internalizing symptoms and higher prosocial behavior ratings (*t*-ES ranged between 1.8 and 3).

**Figure 3.**
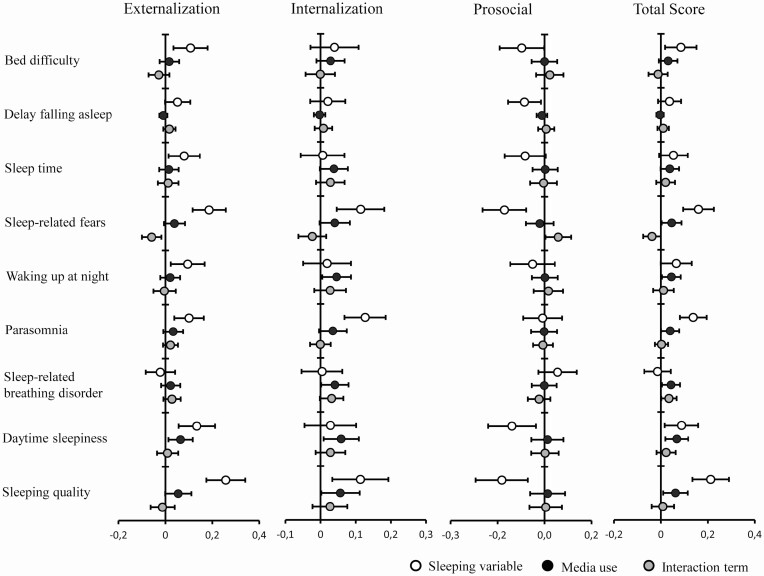
Estimates and 95% CI of standardized beta coefficients of behavioral scores in relation to media use during the weekends.

**Figure 4.**
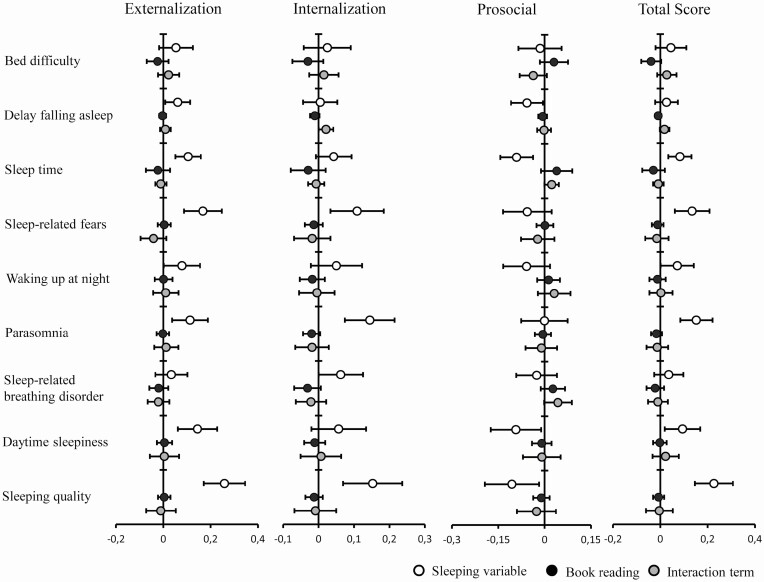
Estimates and 95% CI of standardized beta coefficients of behavioral scores in relation to book reading during the weekends.

For sleep quality we found significant associations between the total CSHQ sleep quality score and prosocial, externalizing and internalizing behavior. Here, especially with internalizing behavior, several CSHQ-subscales such as day time sleepiness, parasomnias, night waking and sleep anxiety were strongly related. For details please see Figures 3 and 4.

All of those associations were apparently stronger when considering media use and book reading during the week but were confirmed during the weekends. The aforementioned results regarding the externalizing and internalizing behavior subscales were confirmed when considering the original SDQ subscales of “conduct”, “hyperactive”, “emotional” and “peers”, respectively.

### Interaction between media use and book reading regarding behavior

The above results were confirmed when looking at the results considering the interaction effect of media watching and book reading on the overall SDQ, internalizing, externalizing and prosocial scores. Specifically, we observed a generalized beneficial association of behavior with book reading and reduced behavioral scores in association with higher exposure to media use. Notably, those analyses highlighted nonsignificance interaction terms between media use and book reading (Table 2).

**Table 2.** Analysis of media watching and book reading interaction effect over the total SDQ score, internalization, externalization and prosocial scores. All models were adjusted fort he overall CSHQ score of the child and by mother’s age, education, anxiety and depression scores defined according to the German version of the Hospital Anxiety and Depression Scale

	Week		Weekends	
Total SDQ	β (stderr)	*t*-value	β (stderr)	*t*-value
Media	0.041 (0.03)	1.38	0.057 (0.03)	1.92
Book	−0.062 (0.02)	−2.53*	−0.045 (0.03)	−1.72^º^
Media* Book	0.021 (0.02)	0.95	0.006 (0.02)	0.27
**Internalization**	β (stderr)	*t*-value	β (stderr)	*t*-value
Media	0.002 (0.03)	0.07	0.037 (0.03)	1.19
Book	−0.05 (0.03)	−1.89*	−0.03 (0.03)	−1.09
Media* Book	0.028 (0.02)	1.17	0.004 (0.02)	0.19
**Externalization**	β (stderr)	*t*-value	β (stderr)	*t*-value
Media	0.044 (0.03)	1.45	0.056 (0.03)	1.83
Book	−0.056 (0.03)	−2.20*	−0.046 (0.03)	−1.72^º^
Media* Book	0.02 (0.02)	0.90	0.014 (0.02)	0.64
**Prosocial**	β (stderr)	*t*-value	β (stderr)	*t*-value
Media	−0.008 (0.03)	−0.25	0.018 (0.03)	0.55
Book	0.052 (0.03)	1.90*	0.017 (0.03)	0.57
Media* Book	0.012 (0.02)	0.49	−0.006 (0.02)	−0.23

***Note*.** Significance levels defined according to *t*-value thresholds 1.64 < |

^º^ *t*-val| < 1.96 (α = 10%); |

*t-val| < 1.96 (α = 5%).

Sensitivity analyses conducted using supplementary adjusting factors did not alter the results. Finally, further sensitivity analyses which compared CSHQ subscale scores between the total sample and the analytical sample did not highlight any possible selection bias due to missing values of the covariates.

## Discussion

Our results indicate significant associations of sleep quality, media use, and book reading on the behavior of children in early childhood. In particular, our results point out that sleep quality is rather related to internalizing than externalizing behavior with parasomnias associated with both externalizing and internalizing behaviors. In contrast, sleep duration, night waking and sleep anxiety was only associated to internalizing behavior. Our results in young children are largely in line with other studies that associated sleep quality to anxiety and depression in school children and adolescents [13, 36, 37]. Moreover, according to our evaluations it seems that book reading and media watching may act independently on child’s behaviors as the interaction between these two factors did not significantly associate with any of the behavior scores.

Considering that sleep quality is associated to both internalizing and externalizing behavior and that numerous aspects of sleep quality in early childhood are associated to media use [32] we should consider that our work add valuable information regarding how media use may represent a direct trigger to internalizing and externalizing behaviors in early childhood. Moreover, our work, along with previous evidence also shows the possible indirect relation of sleeping to internalizing and externalizing behaviors in early childhood [13, 36]. Notably, previous studies evaluated that the association between sleep problems and internalizing and externalizing behaviors in young children is of bidirectional nature [37]. This evidence is not necessarily in contrast with our study. However, according to our results we may hypothesize a more complex pattern for which media use may represents a starting trigger on both sleep quality and behavioral problems which affects each other bidirectionally.

In the present study, we also confirmed that media use in German preschoolers is associated with the total SDQ score and rather with externalizing than with internalizing behavior.

Finally, a major achievement of the current study was the confirmation that book reading could be a healthy entertainment in early childhood resulting in less externalizing and internalizing behavior and in more prosocial behavior [10, 38, 39].

Certain physiological mechanisms have been proposed to support the relation between sleep and behavior. First, sleep is associated to brain functioning and in particular to cerebral glycogen metabolism which, in turn, influences the behavior [40, 41]. Second, a melatonin mediated hormonal pathway of the relation between sleep and behavior has been proposed but is currently under discussion [42–44]. On the one hand, melatonin supplementation has been shown to effectively improve sleeping, while on the other hand, melatonin effect on externalizing behavior has not been studied, yet [45, 46]. However, the lack of the confirmation of a melatonin mediated mechanism influencing the behavior of ADHD children does not necessarily mean that melatonin may not influence externalizing symptoms in healthy children.

Also, psychological mechanisms could be likely involved in the relation between sleep quality and behavior. For example, sleep quality has been demonstrated to influence children’s self-esteem, which, in turn, might lead to internalizing symptoms or could negatively affect prosocial behavior when sleep is impaired [47]. Our results regarding media use and book reading on the behavior in early childhood could be explained by means of the underlying psychological mechanism based on the relation between parents and children. First, during media use or TV watching children are often left alone without their parents being present. This could lead to a lack of parent–child interaction and effective guidance that, in turn, could lead to externalized behavior [3, 48, 49]. Second, book reading, that we suppose being mostly parent assisted for our sample of young children, could improve parent–child interaction, child self-esteem and effective guidance which than might lead to improved problematic and prosocial behavior.

Finally, our results appeared stronger for media use and book reading during the week in comparison to the weekends. This could be due to a possible dose–response mechanism since the sum of the time spent in those activities is larger if we consider the entire week as opposed to the weekends.

The current study has several strengths. First, it was based on a population-based study adopting a prospective design with repeated measures. Second, all psychometric variables were collected using reliable tools validated in the German population. Furthermore, we adopted a rigorous statistical approach using multivariate models taking into account numerous possible confounders to provide reliable results. Last, we provided robust and novel findings regarding the influence of sleep quality, media use and book reading on behavior problems and prosocial behavior of young children.

The study has several limitations, too. The small sample size could have resulted in a high type-II error rate probability, which could have affected the statistical power of the study. As a confirmation, some borderline nonstatistically significant results have been found. Nevertheless, we focused our discussion on results with larger effect sizes providing the reader with a cautious and conservative presentation of the results. We cannot exclude that residual confounding may have limited our results. However, supplementary analyses included more adjusting factors and confirmed the current results. At last, the observational nature of our study, the limited correlation of our variables with observational time and the yearly distance between the time points limited the use of prospective approaches aimed to provide a proper causal interpretation of the results. Finally, the contents of what the children of our sample were watching or reading should have been considered. Unfortunately, such information was not collected.

## Conclusions

Low sleep quality and high levels of media use were negatively associated with the behavior of a sample of young German children. On the contrary, book reading was related to better prosocial behavior and less externalizing and internalizing behavior. Thus, suitable strategies to improve the behavior of young children in Germany include the monitoring of sleep quality, a reduction of media use or the substitution of media use with book reading. In the long run, such low-cost, easy-to-implement and intuitive strategies could reduce economic costs, lower the burden on healthcare and, most importantly, improve the children’s quality of life.
